# Microbial Contamination–Mediated Inflammation Is a Major Contributor of Breast Implant Complications: Prospective Analysis of 631 Samples

**DOI:** 10.3390/jcm15062115

**Published:** 2026-03-10

**Authors:** Celina Kerschbaumer, Konstantin D. Bergmeister, Giovanni Bartellas, Michael Weber, Barbara Ströbele, Melitta Kitzwögerer, Klaus F. Schrögendorfer, Tonatiuh Flores

**Affiliations:** 1 Karl Landsteiner University, Dr. Karl-Dorrek-Straße 30, 3500 Krems, Austriamichael.weber@kl.ac.at (M.W.); barbara.stroebele@stpoelten.lknoe.at (B.S.); tonatiuh.flores@stpoelten.lknoe.at (T.F.); 2 Department of Plastic, Aesthetic and Reconstructive Surgery, University Hospital St. Poelten—NOE LGA, Karl Landsteiner University, Dunant-Platz 1, 3100 St. Poelten, Austria; 3 Clinical Laboratory for Bionic Extremity Reconstruction, Department of Plastic, Reconstructive and Aesthetic Surgery, Medical University of Vienna, 1090 Vienna, Austria; 4 Institute of Hygiene and Microbiology, University Hospital St. Poelten—NOE LGA, Karl Landsteiner University, Dunant-Platz 1, 3100 St. Poelten, Austria; 5 Institute of Clinical Pathology and Molecular Pathology of the Lower Austria Central Region, University Hospitals St. Poelten and Krems—NOE LGA, Karl Landsteiner University, 3100 St. Poelten, Austria

**Keywords:** breast implants, breast implant complications, subclinical infection, chronic inflammation, breast reconstruction, microbial contamination, bacterial contamination

## Abstract

**Introduction:** Breast reconstruction and breast augmentation via silicone breast implants are among the most commonly performed implant surgeries worldwide. However, these surgeries entail notable risks for postoperative implant complications, including implant rupture or capsular contracture. This study investigates the significance of microbial contamination regarding the development of peri-implant inflammation and its impact on implant-related complications. **Methods:** A total of 125 breast implant revisions in 97 patients with a history of breast cancer or prior cosmetic breast augmentation were analyzed at the Clinical Department of Plastic, Aesthetic, and Reconstructive Surgery, University Clinic of St. Poelten, between February 2021 and August 2023. Microbial contamination and subclinical inflammation were assessed using histological and microbiological analysis of implant surfaces and capsules. The implants were grouped by their initial indication for surgery, along with presence of contamination, inflammation, and complications, and then compared using a Chi^2^ test, Fisher’s exact test and two-sided *t*-tests. **Results:** Microbial contamination was found in 27 implants (21.6%), and 58 implants (48.74%) showed histological evidence of inflammation. Peri-implant inflammation was significantly more often observed in contaminated implants (*p* = 0.049). Implants displaying histological signs of peri-implant inflammation showed significantly higher rates of complications, particularly implant rupture (*p* < 0.001 each). In a subgroup analysis, cosmetic patients presented a significantly higher rate of peri-implant inflammation compared to breast cancer patients (*p* < 0.001). Cosmetic patients also showed significantly longer implant inlay times compared to breast cancer patients (14.32 vs. 3.76 years, *p* < 0.001), suggesting that prolonged implant inlay duration may contribute to the occurrence of peri-implant inflammation. **Conclusion:** Subclinical microbial contamination appears to accelerate the inflammatory reactions to silicone implants that subsequently increase the risk for complications and thus surgical removal. Additionally, prolonged implant inlay time seems to be a major independent contributor to chronic, low-grade inflammation, even in the absence of microbial contamination.

## 1. Introduction

Silicone prostheses are the most used implants in plastic surgery due to their use in breast reconstruction and cosmetic augmentation [1,2,3]. Despite rigorous safety testing and improved prosthetic design, a significant number of patients suffer complications such as capsular contracture, implant rupture or pain [4,5,6,7,8]. With prolonged implantation time and a growing population of prosthesis carriers, implant exchange or removal is commonly performed all over the planet [3,8,9]. This results in a high patient burden and increased costs that merit further investigation to tackle this challenge [10,11].

The mechanism of implant damage is multifactorial and not yet fully known. However, peri-implant inflammation and potentially microbial contamination appear to be involved [12,13]. This was recently underlined by linking ruptured implants to contamination with gram-positive bacteria, suggesting that peri-implant stressors significantly compromise implant shells over time [14]. These chronic irritations could even be linked to the development of breast implant-associated anaplastic large cell lymphoma (BIA-ALCL), a rare malignant disease solely affiliated to breast implants [15,16,17,18]. Apart from bacterial contamination, physical stress, or more specifically implant friction, may also be an underlying mechanism causing peri-implant inflammation [19,20,21,22,23].

This research examines the relationship between subclinical infection and chronic inflammation through the integration of histological and microbiological analyses of peri-implant tissues. Its aim is to clarify the influence of bacteria-induced inflammation on the emergence of implant-related complications. To date, there have been no prior studies addressing this specific topic.

## 2. Materials and Methods

### 2.1. Study Design and Patient Analysis

We prospectively analyzed breast surgeries performed from February 2021 to August 2023 at the Department for Plastic, Aesthetic and Reconstructive Surgery of the University Clinic St. Poelten. Included patients underwent breast implant revision due to complications (rupture, capsular contracture, dislocation, perforation, mechanical problems with expander implants, pain, wound healing disorder, deformity) or as the second stage of delayed-immediate breast reconstruction. Our cohort was allocated into two subgroups according to primary surgery indication: reconstruction following a history of breast cancer or aesthetic augmentation.

Preoperative reference swabs were gained from the nipple areolar complex (NAC) and inframammary fold prior to surgical disinfection. Intracapsular swabs and two capsule samples (one for microbiological and one for histological analysis) were collected from each implant intraoperatively. The findings of microbiological and histological analyses were paired with the clinical occurrence of complications. Ethical approval was obtained from the local institutional review board at the Karl Landsteiner University of Health Sciences Krems, Lower Austria, Austria (EK Nr.: 1005/2020).

### 2.2. Perioperative Management

Patients were preoperatively screened for local and systemic inflammation by clinical examination and blood testing. All patients received intravenous single-shot antibiosis preoperatively. An Ampicillin/Sulbactam combination (3g Unasyn^®^, Haupt Pharma Latina S.r.l.; Latina (LT), Italy) or Clindamycin (in case of allergy to penicillin, 600 mg Dalacin^®^, Fareva Amboise; Pocé-sur-Cisse, France) were administered at least 30 min before surgical incision. Patients with a reported history of penicillin-associated vaginal mycosis received Cefuroxim (1.5 g Curocef^®^, GlaxoSmithKline Manufacturing S.p.A.; Verona, Italy). Preoperative surgical disinfection of the operation field was performed following current preoperative antiseptic standardization guidelines using Kodan forte^®^ (Schülke & Mayr GmbH; Vienna, Austria) disinfectant solution.

### 2.3. Operative Procedure

The surgical approach involved excising the old operation scar, with the incision either performed by Lazy-S or Inverted-T in cases of second-stage breast reconstruction or through the inframammary fold in cases of prior aesthetic breast augmentation. Bacteriological intracapsular swabs were gathered immediately after capsule incision using a sterile Amies^®^ agar swab (Copan Italia S.p.A.; Brescia, Italy) without touching the surroundings. In the presence of peri-implant fluid accumulation, cytological aspirates were collected. In cases of macroscopical bloody discoloration, sodium-citrate (3.8%, 1:10) was added to avoid clotting and sample distortion. Afterwards, the old implant was removed manually. Two full-thickness capsule samples measuring at least 1 cm^2^ were collected and stored in Brain Heart Infusion Agar^®^ (Becton Dickinson BDTM; Heidelberg, Germany) for microbiological evaluation or 10% neutrally buffered formalin for pathological examination. All intraoperative samples were collected in a randomized manner. Before inserting the definitive implant, the surgical team changed gloves, and the respective pocket was purged with iodine mixed 1:1 with 10% NaCl. The implant was soaked in antibiotic solution (600 mg of Clindamycin, Dalacin^®^, Fareva Amboise; Pocé-sur-Cisse, France) prior to implantation.

### 2.4. Microbiological Processing

Capsule samples were sent to the Department for Hygiene and Microbiology of the University Clinic of St. Poelten. Following incubation on agar plates, the samples were examined after 24, 48 and 72 h (anaerobe cultures were first examined at 48 h). The respective agar plates were COS (Columbia Agar + 5% sheep blood, Co. bioMerieux; Vienna, Austria) and Voges (chocolate agar—GCII Agar with IsoVitaleX TM, Co., BD; Vienna, Austria). Anaerobe cultures were incubated on Schaedler (Schaedler Agar with Vitamin K1 + 5% sheep blood, Co., BD; Vienna, Austria).

Swab samples were incubated for 48 h on their respective agar plates and examined after 24 and 48 h. The agar plates used for aerobe cultures were COS (Columbia Agar + 5% sheep blood, Co., bioMerieux; Vienna, Austria), CNA (Columbia CNA Agar + 5% sheep blood, Co., BD; Vienna, Austria) and MacConkey (MacConkey II Agar, Co., BD; Vienna, Austria). Anaerobe cultures were incubated on Schaedler (Schaedler Agar with Vitamin K1 + 5% sheep blood, Co., BD; Vienna, Austria) and KV (Schaedler Kanamycin-Vancomycin Agar + 5% sheep blood, Co., BD; Vienna, Austria).

### 2.5. Pathological Processing

Histological samples were sent to the Department for Pathology of the University Clinic of St. Poelten. Following formalin fixation (6–72 h at room temperature), macroscopical examination and preservation for histological processing was performed. Histological cuts of 1–2 μm were prepared, stained with hematoxylin–eosin, and subsequently evaluated. Inflammation characteristics were described in a morphological (acute///chronic///granulomatous) and semiquantitative (low///moderate///high grade) manner.

### 2.6. Statistics and Data Management

All data were processed anonymously. Data protection management complied with Austrian legislation. For statistical analysis, IBM SPSS Statistics for Windows Version 29 (Co., IBM; Armonk, NY, USA) was used. Metric data (Age, BMI) are described using mean ± SD. Nominal data are described as absolute and relative frequencies. Assuming independence of implants, Chi^2^ tests and Fisher’s exact tests were also used to compare

- The two subgroups (reconstruction following a history of breast cancer or aesthetic augmentation) regarding tissue inflammation, microbial contamination and implant complications.
- The complication rates of implants with and without the presence of tissue inflammation for the total group as for each subgroup separately.

Microbial contamination was considered present if one or more microbiological sample from the implant showed bacterial growth. Signs of inflammation were considered positive if the histologically examined capsule sample showed inflammatory cells (lymphocytes, granulocytes, macrophages). *p*-values ≤ 0.05 were considered statistically significant. Due to the relatively small sample size, no multiplicity corrections were performed in order to avoid increasing Type II errors.

## 3. Results

In total, 125 implant revisions in 97 patients fulfilled our inclusion criteria. Patients’ mean age was 51.81 years (std.: 12.81 years). Mean BMI was 25.33 kg/m^2^ (std.: 4.69 kg/m^2^), and mean implant inlay time was 6.95 years (std.: 10.21 years). A total of 85 implants (from 73 patients, 68%) were revised in breast cancer patients, while 40 implants (from 24 patients, 32%) were revised in patients with a history of cosmetic augmentation (Table 1).

In 52% of patients, the revision was performed as the second stage of breast reconstruction. Hereof, 44% were expander-to-definitive implant exchanges, implant removal constituted 34.40% and exchange of definitive implants constituted 12%. Implant-to-free flap surgeries made up 9.60%.

The total number of collected samples was 631, of which 220 were preoperative reference samples. These samples were obtained from the nipple areolar complex and the inframammary fold prior to surgical disinfection. The remaining 411 samples were collected intraoperatively (Table 2).

**Figure 1:**
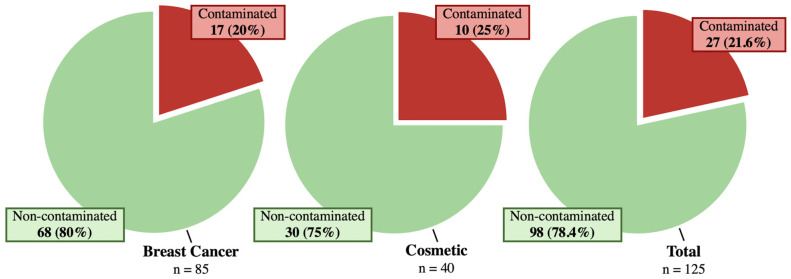
Schematic representation of intraoperative bacterial sampling results, indicating germ-positive implants in red and germ-negative implants in green for each subgroup; (**left**) Breast cancer patients, (**middle**) Cosmetic patients, (**right**) Total study cohort.

Microbial contamination was identified in 27 implants (in 23 patients, 21.6%) (Figure 1). Among all intraoperatively collected microbiological samples, 35 (8.49%) tested positive for bacteria. This included 17 intracapsular swabs (in 16 implants, 12.88%) and 18 capsule samples (in 18 implants, 13.33%).

**Figure 2:**
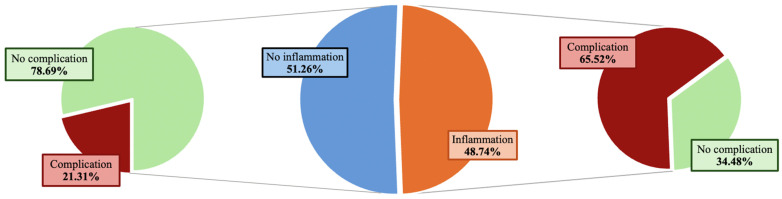
The (**central**) diagram illustrates the distribution of implants with and without histological evidence of inflammation. The (**left**) panel shows complication rates in implants without inflammation, whereas the (**right**) panel shows complication rates in implants with histological signs of inflammation.

The presence of peri-implant inflammation (lymphocytes, granulocytes or macrophages in histological samples) was proven in 58 implants (48.74%), of which 38 implants (65.52%) showed simultaneous complications (Figure 2).

### 3.1. Breast Cancer Patients

Mean age was 53.77 years (std.: ±10.87 years), and mean BMI was 25.06 kg/m^2^ (std.: ±4.47 kg/m^2^). Mean implant inlay time was 3.76 (std.: ±7.19 years) (Table 1). There were 85 implant revisions among 73 breast cancer patients. Second-stage delayed-immediate breast reconstruction was the major revision indication (65 implants, 76.47%). A total of 55 revisions (64.71%) were expander-to-implant exchanges, and 12 (14.12%) were implant-to-free flap procedures. Implant removal was performed in 10 cases (11.76%), and definitive implant exchange in 8 cases (9.41%).

Preoperative radiotherapy was performed on 20 patients (in 20 breasts, 23.53%). All radiotherapy procedures targeted the thoracic wall. Among irradiated patients, Mentor Becker™ expanders (Mentor Worldwide LLC., Irvine, CA, USA) were used in 17 cases (85%), whereas Mentor CPG™ 321 (Mentor Worldwide LLC., Irvine, CA, USA), Eurosilicone™ R022 (GC Aesthetics, Apt, France), and McGhan Allergan 410FX (McGhan, Irvine, CA, USA) were each used once (5%).

Implant-associated complications occurred in 21 implants (24.71%), of which ten showed several complications simultaneously. Rupture was the most common (eight implants, 9.41%), followed by pain (seven implants, 8.24%). Dislocation was seen in five implants (5.88%), and capsular contracture in four implants (4.71%).

The microbiological samples obtained in this group were 92 intracapsular swabs and 87 capsule samples (Table 2). Bacterial growth was seen in 12 swabs (in 11 implants, 12.94%) and 10 capsule samples (in 10 implants, 11.76%), out of a total of 179 intraoperatively obtained microbiological specimens in this group. Overall, 17 implants (in 16 patients, 20%) were contaminated (Figure 1). *S. epidermidis* and *S. lugdunensis* were isolated most frequently (43.75% and 25%) (Table 3). The presence of lymphocytes, granulocytes or macrophages was identified in 28 implants (32.94%), of which 11 implants (39.29%) showed simultaneous complications.

### 3.2. Cosmetic Augmentation Patients

Mean age was 44.22 years (std.: 14.71 years), and mean BMI was 25.99 kg/m^2^ (std.: 5.41 kg/m^2^). Mean implant inlay time was 14.32 years (std.: 12.26 years) (Table 1). The revisions were 40 implants in 24 patients, with implant-associated complications being the main indication (33 implants, 82.5%). The remaining seven implants (17.5%) were revised for prophylactic reasons and to preserve symmetry. In two implants, preliminarily suspected complications (rupture and capsular fibrosis once each) were not verified intraoperatively. Removal alone was performed 33 times (82.5%); seven were implant-to-implant revisions (17.5%).

Implant rupture and capsular fibrosis were most commonly encountered (16 implants each, 40%). Pain was second-most common (15 implants, 35%). Axillary siliconoma occurred with two implants (5%), both of which were ruptured. Multiple complications occurred simultaneously within 16 implants (40%).

The microbiological samples obtained in this group were 42 intracapsular swabs and 45 capsule samples (Table 2). Microbial contamination was seen in five swabs (11.90%) and seven capsule samples (15.56%). These occurred in ten implants (25%) within seven patients (Figure 1). S. epidermidis was isolated most frequently (eight of 13 species, 61.55%) (Table 3). Proof of inflammation within the histological samples (presence of lymphocytes, granulocytes or macrophages) was found in 30 implants (75%), of which 27 implants (90%) presented with concomitant complications.

### 3.3. Microbiological Sample Results

In total, 13 different strains of bacteria were extracted, of which the majority were gram-positive (41 of 43 isolated species, 95.35%). *E. coli* and *P. mirabilis* were the only gram-negative species, occurring once each (Table 3). *S. epidermidis* was identified most frequently (51.16%).

Bacterial contamination was seen in 33 specimens collected from 27 implants (five implants showed multiple germ-positive samples). Implant-related complications and simultaneous bacterial contamination occurred within 15 of 27 implants (55.56%).

Capsular fibrosis was the most frequent complication in contaminated implants, occurring in seven of 27 cases (25.93%). Rupture was second most common (six implants, 22.22%), followed by wound healing disorders (five implants, 18.52%).

**Figure 3:**
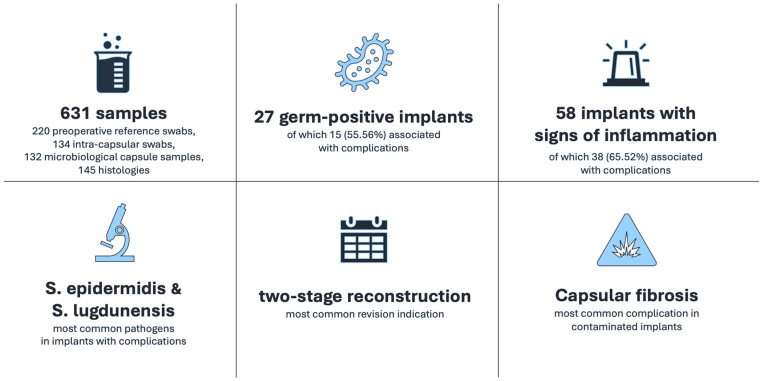
Overview of the main study results. The figure summarizes the total number of collected samples (**upper left**), the prevalence of germ-positive implants (**upper mid**) and the inflammatory findings (**upper right**). Below, the most frequently identified pathogens in implants with complications (**down left**), the most common revision indication (**down mid**), and the predominance of capsular fibrosis among contaminated implants (**down right**) are outlined.

A total of 25 bacterial species were isolated from contaminated implants with the simultaneous appearance of implant complications. *S. epidermidis* was isolated most often (15 samples), followed by *S. lugdunensis* (three samples) (Figure 3).

### 3.4. Statistical Evaluation

Chi^2^ tests and Fisher’s exact tests were used to compare groups of implants with and without bacterial contamination, implant complications and signs of inflammation. Metric group characteristics were compared using two-sample *t*-tests.

Complications occurred significantly more often in patients with signs of inflammation in the overall study cohort (65.5% vs. 21.3%, *p* < 0.001) and within both subgroups (breast cancer: 37.9% vs. 15.4%, *p* = 0.030; cosmetic: 93.1% vs. 55.6%, *p* = 0.020) compared to those without signs of inflammation. Implant rupture was also more prevalent in implants with inflammation in the whole cohort (36.2% vs. 4.9%, *p* < 0.001) and in breast cancer patients (20.7% vs. 3.8%, *p* = 0.024). In the cosmetic subgroup, implant rupture was proportionally more prevalent in the presence of tissue inflammation; however, this was not statistically significant (51,7% vs. 11.1%, *p* = 0.052).

**Figure 4:**
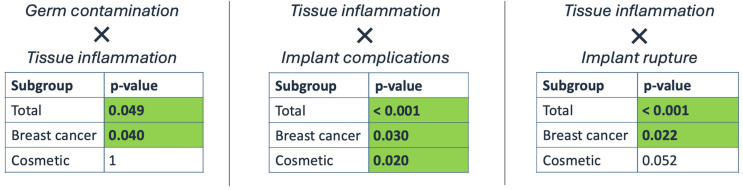
The study variables were compared using a Chi^2^ test of independence [χ^2^ (1, *n* = 125) = x, y, and z] and Fisher’s exact test. The *p*-values reveal significant differences in implant complication rates between implants with and without evidence of inflammation in all study groups (**middle**). Tissue inflammation was observed significantly more frequently in implants with microbial contamination (**left**) and implant rupture (**right**). This proved to be true in the whole study cohort and breast cancer patients.

Comparing implants with and without bacterial contamination, inflammation was observed significantly more often in contaminated implants within the whole cohort (66.7% vs. 44.2%, *p* = 0.049) and the breast cancer group (60% vs. 30.3%, *p* = 0.040). In contrast, within the cosmetic subgroup, the occurrence of inflammation did not differ significantly between contaminated and non-contaminated implants (77.8% vs. 75.9%, *p* = 1) (Figure 4).

Comparing the two subgroups, cosmetic patients exhibited significantly higher complication rates and a higher prevalence of inflammation (*p* < 0.001 each). Implant inlay time was also notably longer in cosmetic patients compared to breast cancer patients (14.32 versus 3.76 years, *p* < 0.001). Preoperative radiotherapy did not affect the occurrence of complications in the breast cancer group (*p* = 0.972). The prevalence of microbial contamination did not significantly differ between the two subgroups (*p* = 0.642).

## 4. Discussion

Breast implant complications represent a major cause of implant revisions [3,24]. Here, secondary contamination is a significant contributor. Dermal germs have been linked to the initiation and progression of secondary peri-implant inflammation [25,26,27]. Despite being mainly attributed to capsular contracture, studies have recently highlighted their significant role in implant rupture [14,28,29]. Prior to implant rupture, deterioration of implant shells over time allows the implant to dispense silicone particles, a process that has previously been linked to carcinogenic risk [19]. BIA-ALCL is a rare malignancy exclusively linked to breast implants, yet its incidence is increasing [23,30,31,32,33]. Although its causes are not fully understood, peri-implant inflammation, as seen in contaminated implant cavities, has been identified as a significant contributor to its pathogenesis [32,34].

Our findings reveal significantly higher frequencies of complications in implants with evidence of peri-implant inflammation in our patients. Across the whole study cohort, implants with microbial contamination showed significantly higher levels of inflammation; however, this was observed only among breast cancer patients in our subgroup analyses. Interestingly, comparing the two subgroups, cosmetic patients showed a higher prevalence of peri-implant inflammation. Additionally, implant inlay was significantly longer in aesthetic patients than in breast cancer patients. These findings suggest that microbial contamination may accelerate peri-implant inflammation and thus complications.

However, additional factors appear to gain inflammatory importance with increasing implant inlay time. The dispensing of silicone particles was proven to start after 8 to 14 years due to the implant shell’s structural fatigue, even without rupture [19]. Silicone gel extrusion has been found to provoke and sustain immune reactions, leading to aberrant immune system activation [19,20]. Long-term mechanical friction caused by movement might further stress the surrounding tissue, leading to accelerated immunologic reactions independent of microbial presence [35].

Substantiating this theory, the occurrence of implant rupture was significantly higher in implants displaying inflammation in the whole study cohort and the breast cancer patients but not in cosmetic patients, thus raising the possibility that chronic, non-microbial inflammation might mediate lower-grade reactions. This could, therefore, exert a less aggressive effect on implant integrity and lead to less structural compromise compared to microbial-mediated inflammation. However, meticulous quantification of both inflammation and microbial burden is necessary to prove this hypothesis in further studies.

In our previous work [14], we proved a significantly higher prevalence of complications in implants with microbial contamination, specifically implant rupture. However, the previous study lacked histological examinations, therefore only allowing us to hypothesize the following pathogenic sequence: microbial contamination initiates inflammatory responses within the peri-implant tissue, which then acts as a mediator for implant complications, mainly implant rupture. This current study confirms our assumption. Additionally, prolonged implant inlay time per se appears to contribute to chronic inflammation, even in the absence of microbial contamination.

Considering our findings, additional preventative strategies to minimize microbial contamination should be addressed, e.g., by supplementing already established protocols. Preoperative skin cleansing with common antiseptic agents could easily be performed by patients. Further, preoperative disinfection of the operating field using standardized antiseptic guidelines could be performed in a prolonged manner. In our department, the patients’ skin is disinfected four times by default, instead of the more common three times.

Additionally, standardized irrigation of the implant cavity with antiseptic solutions (complying with implant manufacturers’ recommendations) ought to eradicate local stressors for contamination. Also, implants are soaked in Clindamycin prior to implantation in our clinic. Modification of antibiotic agents for implant bathing might warrant consideration. Still, further studies are needed focusing on investigating whether antibiogram-guided postoperative antibiotic prophylaxis leads to improved outcomes and reduced evidence of inflammation.

While this study presents significant findings, its interpretation is constrained by the limited number of implants analyzed within the cosmetic group. Future investigations with larger participant cohorts are recommended, with particular attention to the timing of implant complications and the detailed quantitative measurement of both inflammatory responses and microbial load. Such research would provide greater insight into the temporal relationship among microbial presence, inflammation, and complication risk.

## 5. Conclusions

While breast implants are highly resilient, their overall durability remains finite. Because the timing of implant degradation cannot be determined precisely, careful handling of implants is essential. Emphasis should be placed on feasible yet effective measures to reduce the risk of adverse events associated with breast implants, such as maintaining aseptic technique during placement, ensuring adequate postoperative recovery, and minimizing friction on the implant. Although it is not possible to completely eliminate common stresses imposed on breast implants, routine evaluations and diligent aftercare are crucial for optimal outcomes.

## Figures and Tables

**Table 1:** Characteristics of study patients, divided by subgroups: patients with a history of breast cancer (left column), patients with prior cosmetic surgery (middle column) and the overall study cohort (right column).

Patient Characteristics	Breast Cancer	Cosmetic	Total
Age (years)	53.77 ± 10.87	44.22 ± 14.71	51.81 ± 12.81
BMI (kg/m^2^)	25.06 ± 4.47	25.99 ± 5.41	25.33 ± 4.69
Patients included	73 (75.26%)	24 (24.74%)	97 (100%)
Implants revised	85 (68%)	40 (32%)	125 (100%)
Tissue expanders	66 (100%)	0 (0%)	66 (100%)
Definitive implants	19 (32.20%)	40 (67.80%)	59 (100%)
Implant-to-implant revisions	63 (90%)	7 (10%)	70 (100%)
Implant-to-free flap revisions	12 (100%)	0 (0%)	12 (100%)
Implant removals	10 (23.26%)	33 (67.74%)	43 (100%)
Implant inlay time (years)	3.76 ± 7.19	14.32 ± 12.26	6.95 ± 10.21
Preoperative radiation	20 (23.53%)	0 (0%)	20 (16%)

**Table 2:** Samples obtained during surgery within the respective groups. Preoperative microbiological swabs were collected from the NAC and the inframammary fold prior to skin disinfection. Intracapsular microbiological swabs were gathered in a sterile manner immediately after incision of the implant capsule. Two full-thickness capsule specimens measuring at least 1 cm^2^ were excised and submitted for microbiological and histological processing.

Sample	Breast Cancer	Cosmetic	Total
Preoperative swabs	142 (22.50%)	78 (12.36%)	220 (34.87%)
Intracapsular swabs	92 (14.58%)	42 (6.66%)	134 (21.24%)
Microbiological capsule samples	87 (13.79%)	45 (8.72%)	132 (20.92%)
Histopathological capsule samples	90 (14.26%)	55 (8.72%)	145 (22.98%)
Total	411 (65.14%)	220 (34.86%)	631 (100%)

**Table 3:** Distribution of the identified microbial species in the respective subgroups. Note that several germs were found simultaneously in one specimen. The Gram classification is denoted by the symbol in brackets following each bacterial species. (Rounding may result in slight differences in totals).

Bacterial Species (Gram Stain)	Breast Cancer	Cosmetic	Total
*S. epidermidis* (+)	14 (46.67%)	8 (61.55%)	22 (51.16%)
*S. lugdunensis* (+)	8 (26.67%)	1 (7.69%)	9 (20.93%)
*S. capitis* (+)	2 (6.67%)	-	2 (4.65%)
*P. mirabilis* (−)	1 (3.33%)	-	1 (2.33%)
*C. acnes* (+)	1 (3.33%)	-	1 (2.33%)
*E. coli* (−)	1 (3.33%)	-	1 (2.33%)
*C. tuberculostearicum* (+)	1 (3.33%)	-	1 (2.33%)
*S. petrasii* (+)	1 (3.33%)	-	1 (2.33%)
*B. cereus* (+)	1 (3.33%)	-	1 (2.33%)
*A. neuii* (+)	-	1 (7.69%)	1 (2.33%)
*C. avidum* (+)	-	1 (7.69%)	1 (2.33%)
*Propionibacterium* species (+)	-	1 (7.69%)	1 (2.33%)
*S. aureus* (+)	-	1 (7.69%)	1 (2.33%)
Total	30	13	43

## Data Availability

All the data analyzed during the current study are available from the corresponding author upon reasonable request.

## Abbreviations

The following abbreviations are used in this manuscript:

BIA-ALCL	Breast Implant-Associated Anaplastic Large Cell Lymphoma
NAC	Nipple Areolar Complex
